# Targeting Akt by SC66 triggers GSK-3β mediated apoptosis in colon cancer therapy

**DOI:** 10.1186/s12935-019-0837-7

**Published:** 2019-05-09

**Authors:** Yeying Liu, Yuan Huang, Jie Ding, Nannan Liu, Shuang Peng, Jiangang Wang, Feng Wang, Yingjie Zhang

**Affiliations:** 1 Department of Health Management, The Third Xiangya Hospital, Central South University, College of Biology, Hunan University, No. 1, Denggao Road, Changsha, China; 2 Department of Gastroenterology, The Tenth People’s Hospital of Shanghai, Tongji University, Shanghai, China; 3 Department of Emergency Surgery, The Second Military Medical University, Shanghai, China; 4 Shenzhen Institute, Hunan University, Shenzhen, China

**Keywords:** AKT, GSK-3β, Bcl-xL, SC66, Colon cancer

## Abstract

**Background:**

Colon cancer is one of the three common malignant tumors, with lower 5 years survival rate. Akt is an important therapeutic target, while SC66 is a novel allosteric AKT inhibitor, which enhances the therapeutic effect in several types of cancer. However, the molecular mechanisms of targeting AKT by SC66 during colon cancer therapy are not well understood.

**Methods:**

The biological role of GSK-3β in colon cancer growth suppression induced by SC66 was detected in vitro and in vivo. Hoechst 33342 and crystal violet staining were used to determine whether targeting AKT affected apoptosis and cell proliferation. The CCK8 assay was utilized to analyze cell viability. The expression levels of Akt, GSK-3β, Bax, Bcl-xL, p53 and PUMA were measured by immune blotting. Xenograft mouse model was established to study the antitumor effect of SC66 in vivo.

**Results:**

Our results show that SC66 induced significantly colon cancer cell apoptosis, accompanied with Akt inactivation. After AKT inhibition, activated GSK-3β interacted with Bax directly, leading to Bax oligomerization and activation. Knocking down GSK-3β abrogated SC66-triggered Bax activation and apoptosis, which was enhanced by over-expressed GSK-3β. In addition, the expression level of Bcl-xL was down-regulated while p53 had no function during SC66-induced apoptosis. Furthermore, colon cancer growth was suppressed by SC66 therapy in vivo.

**Conclusion:**

Taken together, these data indicated that the novel small molecule AKT inhibitor SC66 shows visible antitumor effects via the AKT/GSK-3β/Bax axis in vitro and in vivo. Our results provide a rational basis for the development of targeting-GSK-3β, which may serve as a potential biomarker and yield meaningful benefits for colon cancer patients in the future.

## Background

Colon cancer is a frequent occurrence in malignancies of digestive tract with an increasing mortality rate, but a low 5-year survival rate [1]. Many significant therapeutic agents over the past decade have been applied for colorectal cancer therapy [2, 3]. However, chemotherapeutic agents gradually turn out to show its drawbacks due to lack of specificity or drug resistances [4, 5]. In this regard, potential drugs with specific target are now being developed for clinical application to cure colorectal cancer patients.

SC66, a novel AKT inhibitor, has shown greater promise than other PIP3/Akt inhibitors against several cancer types, including cervical cancer [6]. As an allosteric AKT inhibitor, SC66 facilitates Akt deactivation more effectively by directly interfering with the PH domain binding to PIP3, and subsequently induces Akt ubiquitination, thereby manifesting a more efficient growth suppression of transformed cells that are associated with a high-level expression of Akt signaling [7]. SC66 also induces alterations in cytoskeleton organization and ROS production, leading to a reduction in total and phospho-AKT levels. SC66 has been demonstrated to inhibit tumor growth of hepatocellular carcinoma significantly via the AKT/mTOR/β-catenin pathway [8]. However, the mechanisms by which SC66 exerts its antitumor activity especially how to induce cell apoptosis are not well-understood.

One of the most typically activated pathways in human colon cancer is the PI3K/AKT signaling, which has been involved in tumor initiation, invasion, vascularization and metastasis [9–12]. It is therefore not surprising that this inappropriately activated signaling contributes to making the AKT important therapeutic target. AKT, also known as protein kinase B (PKB), is a serine/threonine kinase that mediates cell proliferation, protein synthesis, transcription, and apoptosis [13–15]. And its kinase activity is positively mediated by phosphorylation on two key residues Ser473 and Thr308 [16]. Phosphorylation of both residues on is required for maximal AKT1 activation downstream of PI3K [17]. Once activated by a variety of apoptotic stimuli, AKT inhibits the function of the critical tumor suppressor p53 and promotes survival [18]. In addition, several myriad substrates, such as Forkhead Box O3a (FoxO3a), nuclear factor κB (NF-κB) and the mammalian target of rapamycin (mTOR), may be activated via the PI3K/Akt axis [11, 19, 20]. Akt phosphorylates and inhibits several pro-apoptotic gene activities such as Bad, Bim and procaspase 9 [10, 15, 21, 22]. Importantly, activated AKT protects cells from pro-apoptotic stimuli as well as inhibiting GSK-3β [23].

Glycogen synthase kinase-3β (GSK-3β) is a key mediator of apoptosis responding to numerous stimuli [24, 25]. Current studies have demonstrated that active GSK-3β promotes the mitochondrial localization of Bax and induces neuronal apoptosis in response to staurosporine or heat shock [26, 27]. Several Bcl-2 family members are direct substrates/indirect targets of GSK-3β [13]. For example, activated GSK-3β phosphorylates the transcription factor c-Myb, leading to the altered level of Bcl-2 [28, 29]. Pro-apoptotic BH3-only members such as PUMA, Bim, and Bid, indirectly or directly activate the multi-BH domain containing pro-apoptotic signals Bax/Bak, which triggers Bax oligomerization and subsequently causes downstream events including mitochondrial outer membrane permeabilization (MOMP), caspase cascade, and apoptosis [30–33]. Bcl-xL, a critical pro-survival Bcl-2 family member, suppresses apoptosis through the hydrophobic BH3 domain-binding groove of pro-apoptotic signals [34]. Several studies reported certain solid tumor cell lines with low Bcl-xL were sensitive to conventional therapies.

## Materials and methods

### Cell culture and treatment

The human colon cancer cell lines, HCT-116, RKO and DLD1 were obtained from American type culture collection (ATCC). Human colon cancer cell line with p53^−/−^ (HCT-116 p53-KO), and Bax^−/−^ (HCT-116 Bax-KO) were generously provided by Dr Bert Vogelstein (Johns Hopkins University, Baltimore, MD, USA). All the cell lines were routinely cultured in McCoy’s 5A modified medium, supplemented with 10% fetal bovine serum (FBS), penicillin (100 units/ml), and streptomycin (100 mg/ml) in 5% CO2 at 37 °C in humidified incubator. SC66 was purchased from Selleck Chemicals (Houston, TX). The agent of SC66 diluted with DMSO were added in the medium directly. For treatment, the anticancer agent was added in the medium directly before detection.

Transfections were performed with Lipofectamine™ 2000 reagent according to the manufacturer’s protocols (7 sea biotech, Shanghai, China). Cells were transfected with either an empty vector or a constitutively-active Akt expression constructs for 48 h. Then added the related drugs into the culture medium, and replaced with fresh culture medium after 6 h and kept for 12 h. Finally, cells were harvested and proteins were examined to at 24–48 h after transfection.

### Antibodies and reagents

Primary antibodies against p53, Phospho-Akt (S473), Total-Akt, Phospho-GSK-3β, GSK-3β, Bax, Bcl-2, Bcl-XL, Mcl-1, Cox IV, Cleaved-Caspase3 and β-actin were purchased from Cell Signaling Technology. Lipofectamine™ Reagent was purchased from Invitrogen. HRP-conjugated anti-rabbit and or anti-mouse secondary antibodies an ECL-plus kit were from GE Healthcare. SC66 was purchased from Selleck Chemicals (Houston, TX). The oligonucleotide for shGSK-3 was synthesized as 5′-CCGGGTGTGGATCAGTTGGTAGAAACTCGAGTTTCTACCAACTGATCCACACTTTTT-3′. The CCK-8 kit was from 7 sea biotech (Shanghai, China).

### Cell viability and apoptosis assays

Colon cancer cells were cultured in 96-well microplate at a density of 3.5 × 10^3^ cells/well for 24 h. Cell viability was assessed with CCK-8 at indicated time post treatment according to the manufacturer’s instructions. To estimate the viability of the cells, the absorbance of 450 nm (OD450) was measured with a 96-well plate reader (DG5032, Huadong, Nanjing, China).

For analysis of apoptosis by Hoechst 33342 (Invitrogen), colon cells were cultured on the coverslip of a chamber, rinsed with phosphate-buffered saline (PBS) twice, and then added in 1 ml of McCoy’s 5A containing 1 μl Hoechst 33342, incubated at 37 °C with 5% CO_2_ for 15 or 20 min. Apoptosis was assessed through microscopic visualization of condensed chromatin and micronucleation.

For colony formation assays, equal number of cells after different treatments were plated into 6-well plates. Medium was changed every 2 days. Colonies were visualized by crystal violet staining 2 weeks after plat.

### Western blotting

Protein samples were extracted with RIPA buffer (10 mM Tris–HCl (pH 8.0), 1 mM EDTA, 0.5 mM EGTA, 1% Triton X-100, 0.1% sodium deoxycholate, 0.1% SDS. 140 mM NaCl). Equivalent protein samples (30 μg protein extract was loaded on each lane) were subjected to SDS-PAGE on 10% gel or 12% gel. The proteins were then transferred onto PVDF membranes (Millipore) and blocked with 5% non-fat milk for 1 h at room temperature. The membranes, probed with the indicated primary antibodies, were then incubated at 4 °C overnight. Primary antibody was detected by binding horseradish peroxidase (HRP)-conjugated anti-rabbit or anti-mouse secondary antibody with an ECL plus kit. Detection was performed using the Odyssey infrared imaging system (LI-COR, Lincoln, NE). To detect Bax multimerization, purified mitochondrial fractions were cross-linked with DSP [dithiobis (succinimidyl propionate)] (1 mmol/l), followed by Western blotting analysis.

### Flow cytometry

After treatment, human colon cancer cell line with HCT-116 WT, p53^−/−^ (HCT-116 p53-KO), DLD1 and Bax^−/−^ (HCT116 Bax-KO) were suspended in 1 × 10^5^ cells/ml, and 5 μl Annexin V and 5 μl propidium iodide staining solution were added to 100 μl of the cell suspension. Then added 400 μl Binding Buffer to the cell suspension again. After the cells were incubated at room temperature for 10 min in the dark, stained cells were assayed and quantified using a FACSort Flow Cytometer (Beckman Coulter, Brea, CA, USA). Cell debris was excluded from the analysis by an appropriate forward light scatter threshold setting. Compensation was used wherever necessary.

### Co-immunoprecipitation

To detect the interaction between GSK-3β and Bax, about 4 ml of GSK-3β or Bax antibodies respectively were firstly added to 400 ml cell lysates. According to the manufacturer’s protocol, the mixtures were mixed on a rocker at ambient temperature for 2 h. The immunocomplexes were captured by the addition of protein A-agarose (Roche Applied Sciences, Indianpolis, Cat. No. 11 134 515 001) mixed at 1:10 ratio, followed by incubation at ambient temperature for 1 h. The beads were washed by Washing Buffer 1, Washing Buffer 2, Washing Buffer 3 (Roche Applied Sciences, Indianpolis, Cat. No. 11 134 515 001) and then collected by centrifugation at 12,000 rpm for 30 s. After the final wash, the beads were mixed with 60 ml of 2× Laemmli sample buffer, heated at 98 °C for 8 min, and analyzed by Western blotting using GSK-3β, Bax6A7 or Bax antibody (Cell Signaling Technology, Shanghai, China).

### Xenograft mouse model and treatment

Female 5-week-old nude mice (Vital River, China) were housed in a sterile environment with micro isolator cages and allowed access to water and chow ad libitum. HCT-116 WT was harvested, and 1 × 10^6^ cells in 0.2 ml of McCoy’s 5A modified medium were implanted subcutaneously into the back of athymic nude female mice. Mice were treated with daily with SC66 at 25 mg/kg by i.p. injection every other 3 days for 15 days, whereas the control mice were administered vehicle. Volume was calculated by the formula of 0.5 × length × width^2^. Mice were euthanized when tumors reached ~ 1.0 cm^3^ in size. Tumors were dissected and fixed in 10% formalin and embedded in paraffin. All mice were housed and maintained under specific pathogen-free (SOPF) conditions. All animal studies were in accordance with institutional guidelines and approved by the Use Committee for Animal Care.

### Statistical analysis

Statistical analyses were performed using GraphPad Prism V software. All assays were repeated independently more than three times. Data are represented as mean ± SEM in the figures. P values were calculated using the Student’s paired t-test.

## Results

### SC66 suppressed proliferation in various colon cancer cells by targeting Akt

**Fig. 1:**
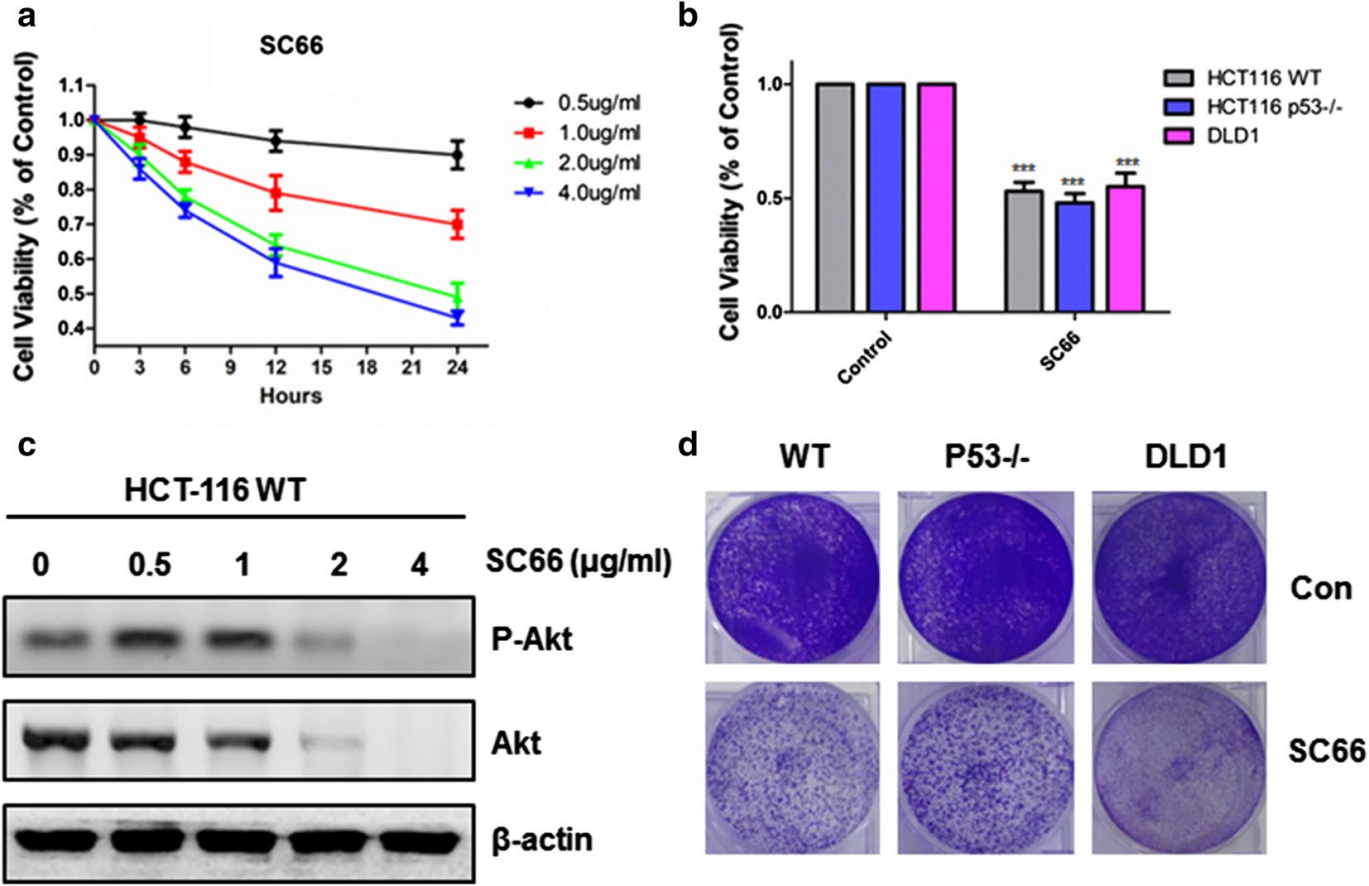
SC66 inhibited colon cancer cell proliferation in vitro. **a** Cell viability of HCT-116 was detected using cell counting kit-8 after 0, 0.5, 1, 2 or 4 μg/ml SC66 treatment at indicated time. Data represent the mean ± SEM of four independent experiments. **b** Cell viability was analyzed in various colon cancer cells after 2 μg/ml SC66 treatment for 24 h. Data represent the mean ± SEM of four independent experiments. **c** Western blotting analysis of P-Akt and Akt expression in HCT-116 WT treated with 2 μg/ml SC66 treatment for 24 h. **d** Colony formation was analyzed by crystal violet staining in HCT-116 WT, HCT-116 p53^−/−^ and DLD1 cells following 2 μg/ml SC66 treatment for 24 h. Similar results were obtained from three independent experiments

To investigate how SC66 influences tumor growth, cell viability was detected by CCK-8 in HCT-116 after 0.5–4 μg/ml SC66 treatment for 24 h. Our results indicated that cell viability decreased significantly with an increasing dose treatment (Fig. 1a), suggesting SC66 inhibited cell proliferation in a dose-dependent manner. SC66, a novel allosteric AKT inhibitor, directly interacts with AKT and facilitates its deactivation and ubiquitination [7]. To study whether the cytotoxic effects of SC66 were associated with changes in AKT signaling, western blotting analysis was detected after SC66 treatment. As shown in the Fig. 1c, the results suggested that activated Akt was over expressed in HCT-116 and slightly increased the phosphorylation level of AKT after 0.5 and 1 μg/ml SC66 treatment, whereas the phosphorylation level of AKT were decreased significantly at a dose of 2 μg/ml SC66 treatment. At the same time, analysis of total AKT protein levels revealed an obvious decrease at a dose of 2 μg/ml SC66 in HCT-116.

Next, to determine a potential role of p53 in the process, HCT-116 WT, p53^−/−^ and DLD1 (p53 mutant) cells were treated with 2 μg/ml SC66 for 24 h. As a result, each cell line had an obvious decrease in cell viability observed after SC66 treatment, indicating p53 was dispensable (Fig. 1b). Next, as shown in the Fig. 1d, colony formation assay was performed in HCT-116 WT, p53^−/−^ and DLD1 cells, which mimics the clonogenic survival of cancer cells in a solid tumor environment. SC66 displayed a potent inhibition of colony forming capacity, which further confirmed the previous results and got a similar conclusion. Taken together, all these data above indicate that SC66 induces AKT phosphorylation and degradation in a p53-independent manner, leading to inhibition of colon cancer cell proliferation.

### SC66 induced intrinsic mitochondrial apoptosis in a p53-independent manner

**Fig. 2:**
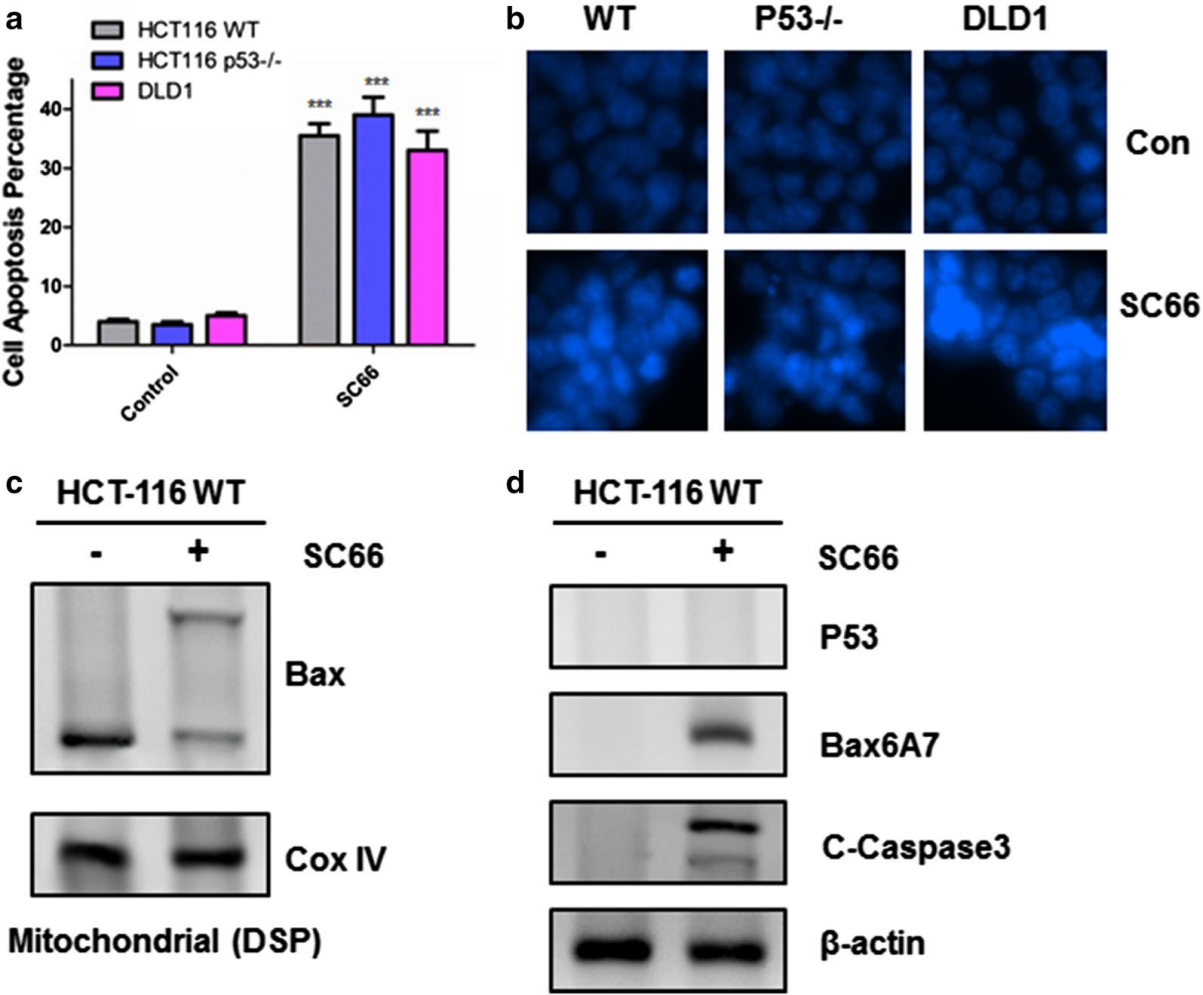
SC66 triggered cell apoptosis in a p53-independent pathway. **a** Cell apoptosis was analyzed by fluorescence-activated cell sorting (FACS) analysis after HCT-116 WT, HCT-116 p53^−/−^ and DLD1 cells were treated with 2 μg/ml SC66 for 24 h. The percentage of apoptotic cells were calculated from fluorescence activated cell sorting (FACS) analysis. Data represent the mean ± SD of three independent experiments. ***P < 0.001 vs untreated control cells. **b** Hoechst 33342 morphological examination of apoptosis in HCT-116 WT, HCT-116 p53^−/−^ and DLD1 cells were treated with 2 μg/ml SC66 and incubated for 24 h, then stained with Hoechst 33342. **c** Bax multimerization in mitochondria fraction was detected by Western blotting after DSP cross-link. **d** Western blotting show the expression of p53, PUMA, Bax6A7 and cleaved Caspase 3 in HCT-116 with 2 μg/ml SC66 for 24 h. Similar results were obtained from three independent experiments

To determine whether SC66 kill colon cancer cells through initiating the process of apoptosis, FACS analysis was performed in HCT-116 WT, p53^−/−^ and DLD1 cells (Fig. 2a). The results indicated that p53 deficiency or p53 mutation had nearly no influence on SC66-induced apoptosis, compared to expressing p53 normally in HCT-116 WT. The morphology of apoptosis was investigated by using Hoechst 33342 staining, and similar trends were obtained. Besides, in the HCT-116 cells, Bax activation and cleavage of Caspase 3 were markedly detected (Fig. 2d), meaning SC66 induced apoptosis through the intrinsic mitochondrial pathway. Interestingly, western blotting analysis has shown that SC66 treatment did not cause p53 activation at all (Figs. 1c and 2d), suggesting Akt inactivation was independent of p53 responding to SC66 stimulation. To further confirm whether SC66-triggered Bax activation, Bax oligomerization in mitochondria was also detected (Fig. 2c). In short, these results revealed that SC66 triggers apoptosis in a p53-independent manner.

### Targeting Akt activated GSK-3β and down-regulated Bcl-xL

**Fig. 3:**
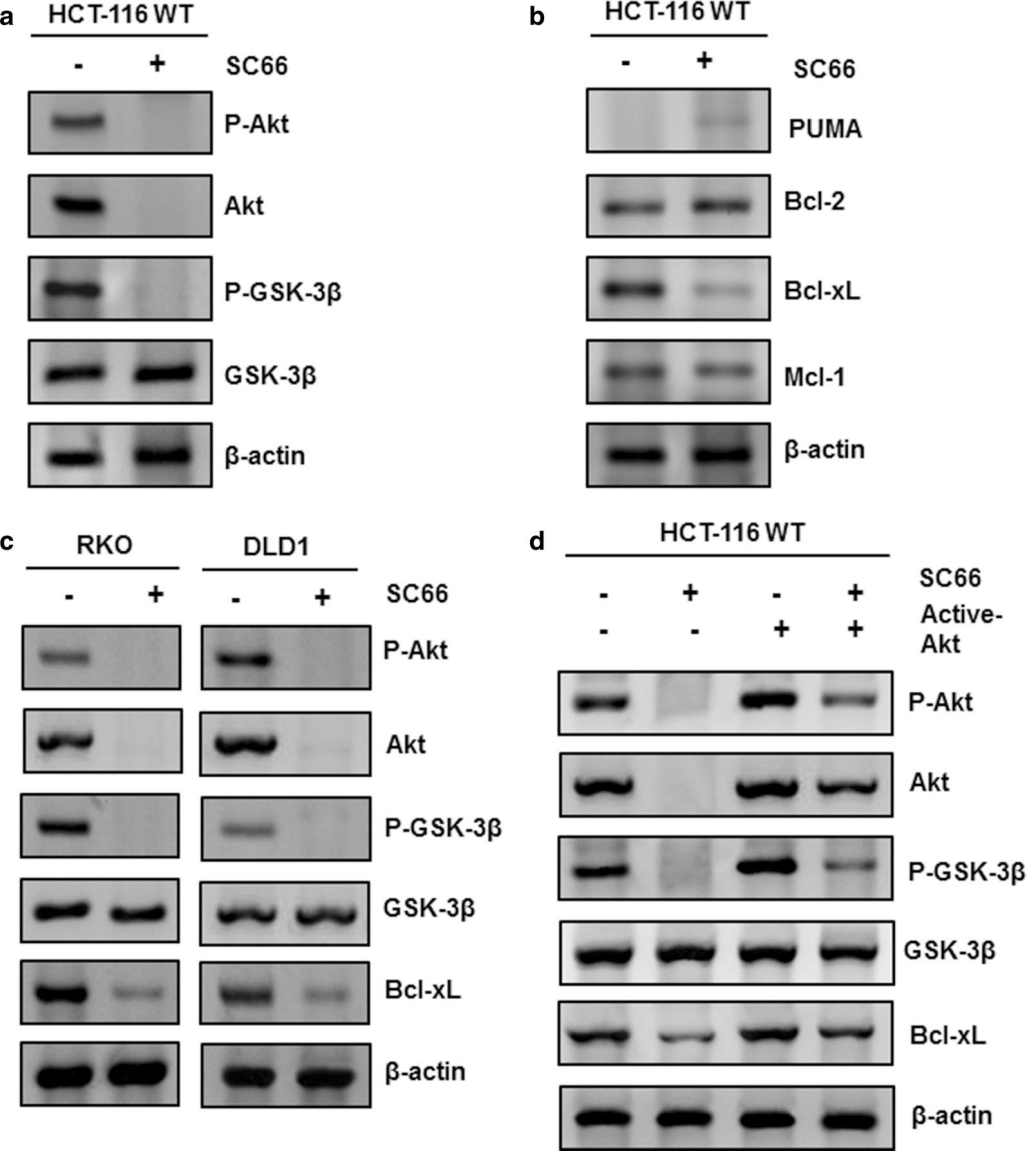
SC66 suppressed cell growth by inhibiting AKT, activating GSK-3β**/**Bax and diminishing Bcl-xL. **a** P-AKT, AKT, P-GSK-3β and GSK-3β expression were analyzed by western blotting in HCT-116 at 30 min after 2 μg/ml SC66 treatment. **b** Puma, Bcl-2, Bcl-xL and Mcl-1 were detected in HCT-116 cells after SC66 for 24 h. **c** AKT, GSK-3β and Bcl-xL, expressions were analyzed by western blotting in RKO and DLD1 after 2 μg/ml SC66 treatment for 24 h. The levels of P-Akt (S473), Akt P-GSK-3β, GSK-3β and Bcl-xL were analyzed by Western blotting. Similar results were obtained from three independent experiments. **d** HCT-116 WT were transfected with either empty vector or a constitutively-active Akt expression constructs for 48 h, then 2 μg/ml SC66 was added into the culture medium and kept for 12 h, finally cells were harvested to detect the results. The levels of P-Akt (S473), Akt P-GSK-3β, GSK-3β and Bcl-xL were analyzed by Western blotting. Similar results were obtained from three independent experiments

To explore the mechanism through which SC66 induce cell apoptosis, western blotting assays were performed. Previous data indicated that AKT may activate a classical down-stream target GSK3β. To confirm the modulation of AKT and its down-stream signaling, we evaluated the phosphorylation levels of AKT and GSK-3β proteins. Treatment with 2 μg/ml SC66 reduced the expression of phospho-AKT and total AKT, and subsequently resulted in the dephosphorylation of GSK-3β in HCT-116 (Fig. 3a). These data mean AKT blockade leads to the reduction of phospho-GSK-3β level, suggesting SC66 accelerates the activation of GSK-3β through AKT inhibition.

Our previous results have proved that SC66 triggered cell apoptosis through Bax oligomerization and its activation (Fig. 2c, d). As shown in Fig. 3b, other Bcl-2 family members were detected. The expression of pro-apoptosis proteins such as PUMA and Bcl-2 did not alter following SC66 treatment. In contrast, SC66 treatment did reduce Bcl-xL protein level obviously and also decrease Mcl-1 protein level slightly. Next, we demonstrated that SC66 activated GSK-3β/Bax and diminished Bcl-xL in other colon cancer cells including RKO and DLD1 cells regardless of the p53 status (Fig. 3c). As a result, AKT was overexpressed in HCT-116 and significantly abolished the SC66-induced decrease of p-AKT (Ser473) and p-GSK-3β levels, as well as the increase of Bcl-xL level (Fig. 3d). As shown in Fig. 3c, d, SC66 inhibited Akt phosphorylation and total-Akt expression, however, did not affect the expression of total-GSK-3β. These results strongly indicate that Akt inactivation after SC66 treatment activates GSK-3β/Bax, and simultaneously inhibits Bcl-xL expression in colon cancer cells regardless of p53 status.

### GSK3β interacted with Bax directly which is indispensable for Bcl-xL down-regulation and SC66-induced apoptosis

**Fig. 4:**
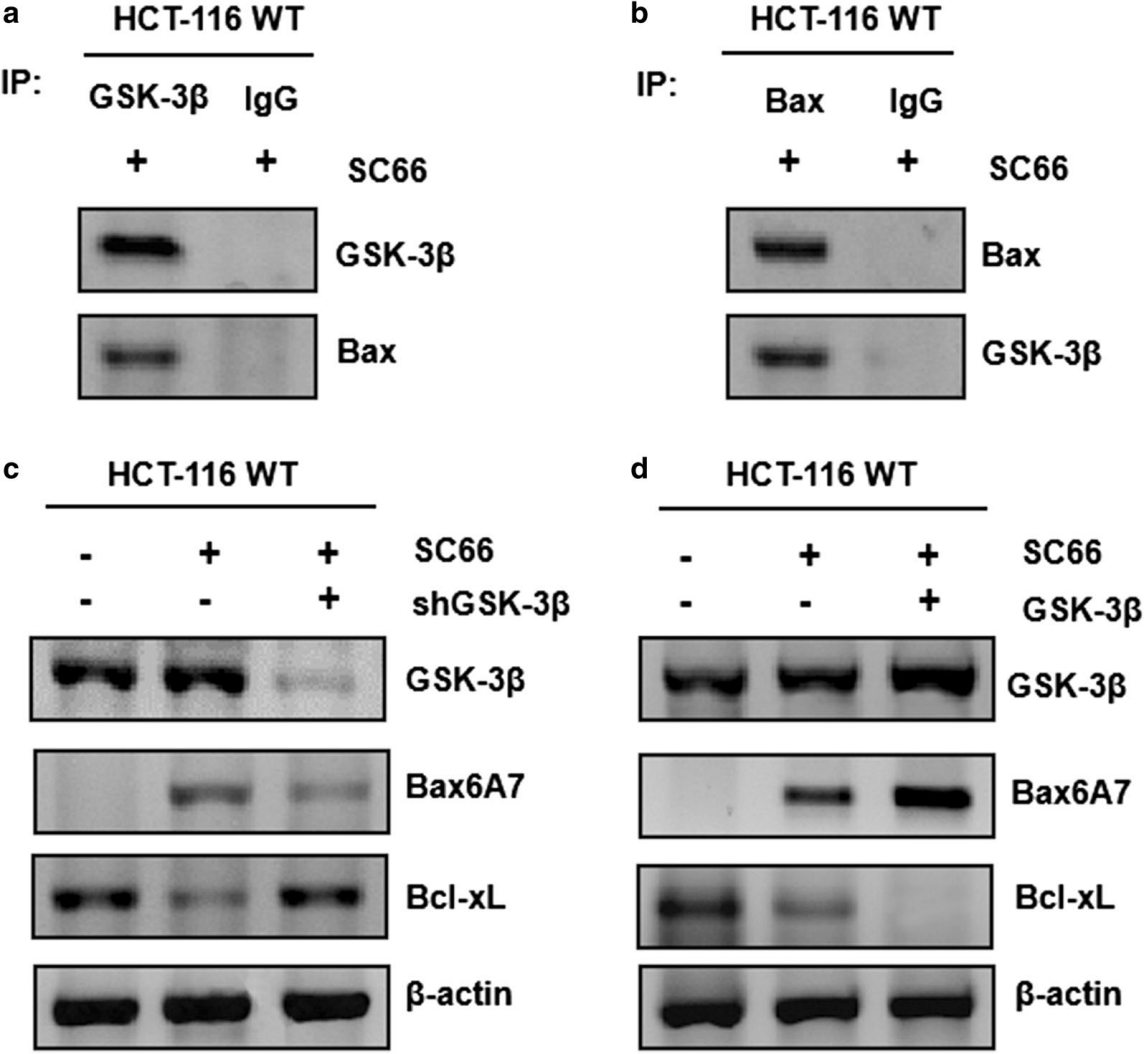
GSK-3β activated Bax directly and inhibited Bcl-xL indirectly, leading to SC66-induced apoptosis. **a**, **b** The interaction between GSK-3β and Bax increased after SC66 treatment. Co-immunoprecipitation with an anti-GSK-3β antibody or anti-Bax antibody was used to pull down GSK-3β or Bax. **a** Western blotting for Bax showed the amount of Bax binding to GSK-3β. **b** Western blotting for GSK-3β showed the amount of GSK-3β binding to Bax. IgG was used to as a control for the GSK-3β-specific antibody. **c**, **d** The effect of GSK-3β knockdown or GSK-3β over-expression on colon cancer cells. **c** Bax6A7, Bcl-xL and GSK-3β were detected in HCT-116 WT following the treatment of 2 μg/ml SC66, with GSK-3β knockdown or not. Similar results were obtained from three independent experiments. **d** HCT116 WT were transfected with either empty vector or a constitutively-active GSK-3β expression constructs for 24 h, following 24 h of 2 μg/ml SC66 treatment. The levels of Bax6A7, Bcl-xL and GSK-3β were analyzed by Western blotting. Similar results were obtained from three independent experiments

Our previous studies have proved that inactivated GSK-3β suppresses Bax translocation via the PI3K/AKT/GSK-3β axis after LPLI treatment [35]. In this study, to test whether GSK-3β binds to Bax following SC66 treatment, the endogenous GSK-3β and Bax complex was further confirmed by reciprocal immunoprecipitation (Fig. 4a, b), meaning GSK-3β binds to Bax directly after 2 μg/ml SC66 treatment. To further confirm whether GSK-3β was indispensable in this process, GSK-3β was knocked down by using shRNA in HCT-116. Next, the activated/pro-apoptotic form of Bax was detected by using a conformation-specific anti-Bax monoclonal antibody (6A7). As shown in the Fig. 4c, knocking down GSK-3β effectively eliminated SC66-triggered Bax activation and Bcl-xL diminution in the presence of SC66.

Furthermore, the results of Fig. 4c, d indicated that GSK-3β knockdown and overexpression is effective. Taken together, the results demonstrated over-expression of constitutively active GSK-3β in HCT-116 markedly enhanced SC66-induced the activation of Bax, as well as the diminution of Bcl-xL, suggesting GSK-3β also activates Bax translocation indirectly by inhibiting Bcl-xL and subsequently modulating the interaction between Bax and Bcl-xL after SC66 stimulation. Together, these data show that GSK-3β activates Bax directly and inhibits Bcl-xL simultaneously through the mitochondrial pathway, during which GSK-3β is necessary for the proapoptotic effect of SC66 in colon cancer cells.

### SC66 induced apoptosis via AKT/GSK-3β/Bax axis

**Fig. 5:**
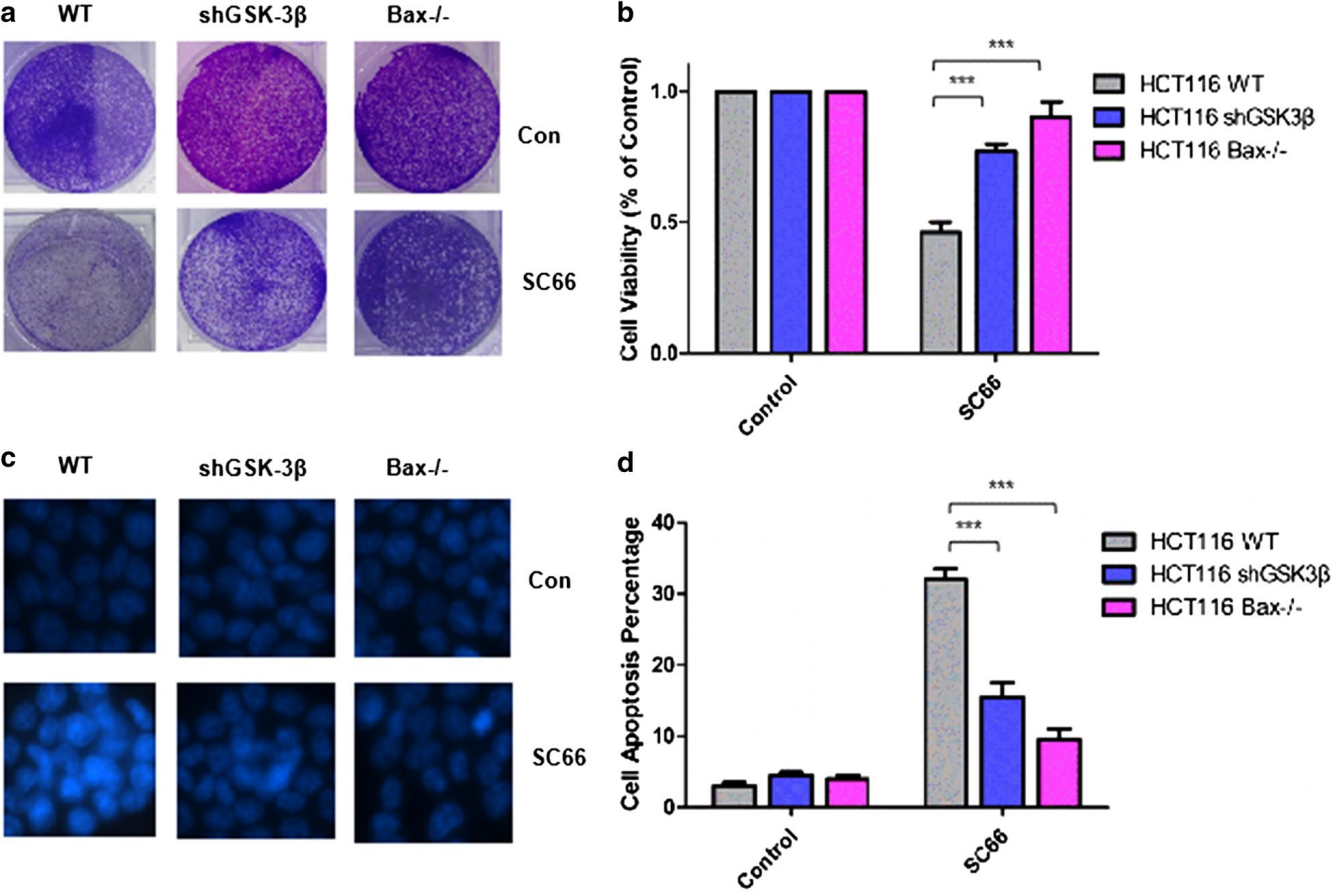
SC66 induced AKT/GSK-3β/Bax-dependent apoptosis. **a** Colony formation was analyzed by crystal violet staining in HCT-116 WT, GSK-3β knockdown or Bax^−/−^ after 24 h of 2 μg/ml SC66 treatment. **b** Cell viability was analyzed using Cell Counting Kit-8 after SC66 treatment in HCT-116 WT, GSK-3β knockdown or Bax^−/−^. Data represent the mean ± SEM of four independent experiments. **c** Hoechst 33242 morphological examination of apoptosis in HCT-116 WT, GSK-3β knockdown or Bax^−/−^ cells were treated with 2 μg/ml SC66 and incubated for 24 h, then stained with Hoechst 33342. **d** Cell apoptosis was analyzed by fluorescence-activated cell sorting (FACS) analysis after in HCT-116 WT, GSK-3β knockdown or Bax^−/−^ were treated with 2 μg/ml SC66 for 24 h. The percentage of apoptotic cells were calculated from FACS analysis. Data represent the mean ± SD of three independent experiments. ***P < 0.001 vs untreated control cells

To explore the essential role of Bax activation in GSK-3β-mediated apoptosis, wild-type or Bax^−/−^, HCT-116 cells were treated with SC66 for 24 h. Meanwhile, as a direct upstream target of Bax, GSK-3β was deleted by shGSK-3β in HCT-116 cell to evaluate its function. As shown in Fig. 5a, colony formation assay was performed. As a result, GSK-3β deletion evidently induced cell growth inhibition, while Bax deficiency almost completely inhibited. Cell viability detection gave us the similar trends (Fig. 5b). Next, the morphology of apoptosis was performed by using Hoechst 33324 staining (Fig. 5c). The results demonstrated that both GSK-3β deletion and Bax deficiency played a significant role in suppressing cell apoptosis. Flow cytometry experiments were also performed, which further confirmed the previous results and got a similar conclusion (Fig. 5d). In short, these results reveal that AKT inhibition promotes the process of SC66-induced cell apoptosis through GSK-3β/Bax-dependent pathway.

### SC66 suppressed tumor growth in vivo

**Fig. 6:**
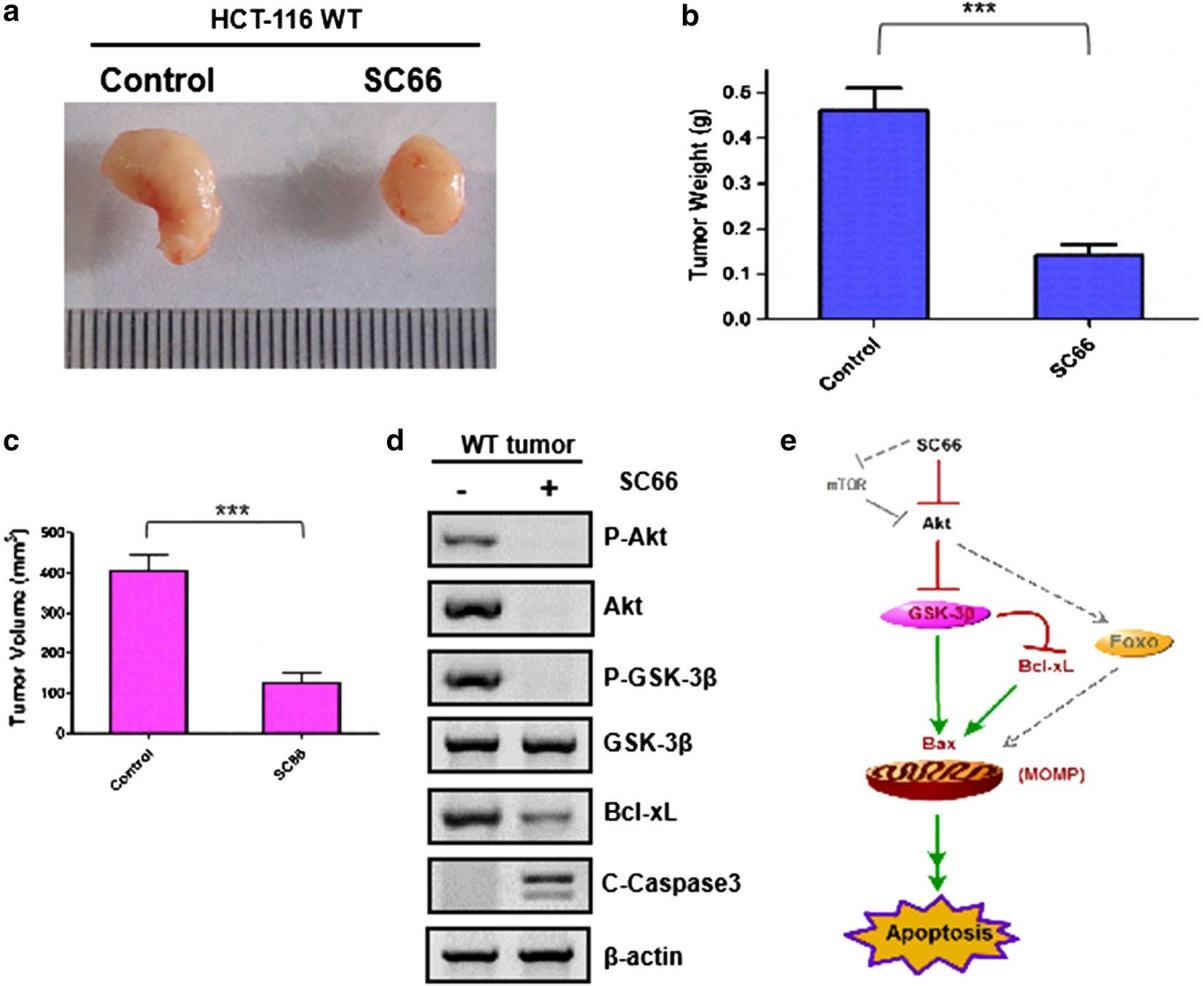
SC66 exhibited the anti-tumor effects in vivo. Nude mice were injected s.c. with 1 × 10^6^ HCT-116. Once the tumor was measurable, mice were treated daily with SC66 at 25 mg/kg by i.p. injection every other 3 days for 15 days. **a** Representative tumors at the end of the experiment. (the most significant group of three). **b**, **c** Tumor weight (**b**) and tumor volume at (**c**) indicated time points after treatment was calculated (n = 3 per group). Statistical significance is indicated for the comparison of SC66-treated tumor. Data represent the mean ± SEM of four independent experiments. ***P < 0.001 vs. WT. **d** The levels of P-Akt, total-Akt, P-GSK-3β, total-GSK-3β and Bcl-xL were analyzed in representative tumors by Western blotting analysis. Similar results were obtained from three independent experiments. **e** Schematic representation of SC66 induced apoptotic pathway

To demonstrate the effectiveness in vivo of SC66 on CRC, we established subcutaneous tumors using HCT-116 WT cells in xenograft mice models. When solid tumors became palpable, at a size of about 150 mm^3^, nude mice were randomly divided into two groups with 3 mice in each group. The treated group received SC66 at 25 mg/kg by i.p. injection every other 3 days for 15 days (PBS was used as the untreated control). As observed in Fig. 6a, the growth of parental tumor was significantly inhibited by about 65% following SC66 stimulation, meaning a satisfactory level of drug cytotoxicity. Quantitative analysis on the body weight and volume of tumors obtained similar trends (Fig. 6b, c). The levels of P-Akt, total-Akt, P-GSK-3β, total-GSK-3β and Bcl-xL were analyzed and detected in representative tumors’ samples. The Western blotting analysis were also performed to further confirm the previous results and got a similar conclusion (Fig. 6d). All these data suggested SC66 displayed a significant tumor growth reduction.

## Discussion

Colorectal cancer remains the third most commonly diagnosed malignancies in the worldwide, which has been estimated about 1.2 million new cases and almost 600,000 deaths annually [36, 37]. Resistance to conventional systemic frontline therapies is a big hurdle in treating colon cancer and reducing serious side-effects due to lack of specificity such as 5-FU, leucovorin, oxaliplatin and irinotecan [38–40]. As we all know, the aberrant activation of AKT/PKB pathway in a variety of cancers including colon cancer is crucial in the proliferation, resistance to apoptosis, metabolism and angiogenesis. AKT mediates cell survival and death by directly and indirectly phosphorylating pro-apoptotic Bcl-2 family proteins or regulating transcriptional factors (for example, FoxO3a, NF-κB, p73 and p53) [15, 19, 41–43]. Therefore, novel therapeutic strategies are needed to advance for cancer patients, suggesting designing of new drugs targeting alternative AKT signaling may be more attractive and appeal for therapeutic intervention.

Historically, several available molecules inhibiting AKT are categorized into three groups including ATP-competitive inhibitors, phosphatidylinositol analogs, and allosteric inhibitors [17]. Novel agents such as NVP-BEZ235 and MK-2206, are now in preclinical development [44–47]. More recently, SC66 is a potent and highly selective allosteric pan AKT inhibitor, which facilitates AKT ubiquitination and further inhibits glucose uptake in vitro and in vivo. Recent studies have reported that SC66 has shown a strong anti-tumor activity, including hepatocellular carcinoma and cervical cancer with PIK3CA R88Q and PTEN R233* mutation [6, 8]. However, the antitumor effects of SC66 and the underlying mechanism in colon cancer still remain to be elucidated.

In the current study, we investigated the potential antitumor activity of SC66 on colon cancer cell lines, showing that colon cancer growth was effectively suppressed after SC66 simulation (Figs. 1, 2, 5 and 6). Next, we further studied the precise mechanism of SC66 induced-apoptosis. Using multiple cell lines including WT, p53 mutant or knockout cells, we demonstrated that SC66 induced apoptosis through p53-independent pathway for the first time (Figs. 1, 2 and 3).

Previous reports from our laboratory have demonstrated that low-power laser irradiation exerts protective effects in preventing cell apoptosis by activating AKT and inactivating GSK-3β [35]. Current studies have demonstrated oligo-porphyran offers a neuroprotective treatment for Parkinson’s disease via PI3K/Akt/GSK-3β pathway, with changes in the Bax/Bcl-2 ratio [48]. However, some studies showed that activated GSK-3β promoted Bax activation in a p53-dependent pathway [49]. Our results showed that AKT activity was highly inhibited in various colon cancer cell lines after SC66 treatment, suggesting AKT signal is independent of p53. The canonical pathway of GSK-3β activation is mediated by NF-κB phosphorylation, for example, in response to sorafenib [42]. Notably, we confirmed SC66 showed greatly suppression of AKT activity and induced apoptosis through the significant factor GSK-3β, but not depend on the important factor such as p53 (Figs. 2, 3).

AKT inhibition led to activation of GSK-3β, that promoted Bax translocation by directly binding to Bax, simultaneously decreasing Bcl-xL, and subsequently triggering the mitochondrial apoptosis (Figs. 3, 4, 5). In addition, GSK-3β/Bax axis is indispensable for using SC66 to treat colon cancer (Figs. 4, 5, 6). The influence of Bax deficiency on SC66 induced-apoptosis is inferior to that of GSK-3β deletion in HCT-116. These results above indicated that SC66 regulated cell survival and apoptosis via the AKT/GSK-3β/Bax pathway, which is distinct from previous reports involved the PI3K/Akt/mTOR or PI3K/PKB/Egr-1 pathway [6, 50].

Research has identified that SC66 interferes with the PH domain binding to PIP3 and directly enhances Akt ubiquitination and its deactivation in both HeLa and HEK293T cells [7]. Recent report claims that SC66 had significant anti-tumor effects on hepatocellular carcinoma cells through producing ROS, subsequently inducing anoikis-mediated cell death and inhibiting the AKT signaling pathway [8]. And the novel Akt inhibitor SC66 directly deactivated Akt and facilitated the activation of Foxo, eventually triggering apoptosis. Some studies also indicated that SC66 effectively inhibited the phosphorylation levels of AKT through disruption of mTOR signaling, and therefore contributes to decrease the expressions of p-GSK-3β and p-FOXO1 in human cervical cancer [6]. Further study in this area may be instructive in different Akt inhibitors, dual PI3K/mTOR inhibitors or other multi-kinase inhibitors.

Previous studies also demonstrated Akt inhibition by pazopanib, ipatasertib or NVP-BEZ235 induced PUMA-dependent apoptosis in colon cancer through activating FoxO3a transcriptionally [32, 33, 51]. Besides, the results indicated that Mcl may also play some roles while Bax and Bcl-xL seem to be the key regulators of mediating cell apoptosis (Fig. 3). A recent study indicated that regorafenib induced colon cancer cell apoptosis via GSK-3β/Mcl-1 axis [52]. Therefore, it seems a link between GSK-3β and Mcl is complicated, which is worth to be further studied.

In conclusion, we provided the first evidence that SC66 exerts its antitumor effects through GSK-3β/Bax pathway through AKT inhibition and initiates apoptosis through the mitochondrial pathway, which is p53-independent. GSK-3β deletion or Bax deficiency abrogated SC66-induced apoptosis and promoted colon cell survival. Together with the data from xenograft mice in vivo, we highlight the novel AKT inhibitor SC66 may function as a promising and potential therapeutic drug for colon cancer treatment, providing the rationale for clinical application in the future.

## Conclusion

Previous reports from our laboratory have shown that low-power laser irradiation (LPLI) inactivates GSK-3β and inhibits apoptosis via the PI3K/Akt pathway [35]. In this study, the anti-tumor effect of SC66 in colon cancer and molecular mechanism behind were clarified. Our results demonstrated that the novel small molecule AKT inhibitor SC66 suppressed colon cancer cell growth through the AKT/GSK-3β/Bax axis irrespective of p53 status. GSK-3β deletion resulted in resistance to SC66-induced apoptosis, which is through the mitochondrial pathway. SC66 also suppressed tumor growth in vivo (xenograft mice). Together, these results indicated the therapeutic response to SC66 is through Akt/GSK-3β/Bax axis, which served GSK-3β as a potential biomarker for colon cancer therapy.

## Abbreviations

GSK-3β: glycogen synthase kinase-3β
Bax: Bcl-2-associated X protein
CRC: colorectal cancer
CCK-8: cell counting kit-8
IHC: immunohistochemistry
FACS: fluorescence activated cell sorting
